# Tweets on the Road

**DOI:** 10.1371/journal.pone.0105407

**Published:** 2014-08-20

**Authors:** Maxime Lenormand, Antònia Tugores, Pere Colet, José J. Ramasco

**Affiliations:** Instituto de Física Interdisciplinar y Sistemas Complejos IFISC (CSIC-UIB), Palma de Mallorca, Spain; University of Maribor, Slovenia

## Abstract

The pervasiveness of mobile devices, which is increasing daily, is generating a vast amount of geo-located data allowing us to gain further insights into human behaviors. In particular, this new technology enables users to communicate through mobile social media applications, such as Twitter, anytime and anywhere. Thus, geo-located tweets offer the possibility to carry out in-depth studies on human mobility. In this paper, we study the use of Twitter in transportation by identifying tweets posted from roads and rails in Europe between September 2012 and November 2013. We compute the percentage of highway and railway segments covered by tweets in 39 countries. The coverages are very different from country to country and their variability can be partially explained by differences in Twitter penetration rates. Still, some of these differences might be related to cultural factors regarding mobility habits and interacting socially online. Analyzing particular road sectors, our results show a positive correlation between the number of tweets on the road and the Average Annual Daily Traffic on highways in France and in the UK. Transport modality can be studied with these data as well, for which we discover very heterogeneous usage patterns across the continent.

## Introduction

An increasing number of geo-located data are generated everyday through mobile devices. This information allows for a better characterization of social interactions and human mobility patterns [1], [2]. Indeed, several data sets coming from different sources have been analyzed during the last few years. Some examples include cell phone records [3]–[16], credit card use information [17], GPS data from devices installed in cars [18], [19], geolocated tweets [20]–[25] or Foursquare data [26]. This information led to notable insights in human mobility at individual level [5], [24], but it makes also possible to introduce new methods to extract origin-destination tables at a more aggregated scale [7], [13], [25], to study the structure of cities [16] and even to determine land use patterns [11], [12], [15], [25].

In this work, we analyze a Twitter database containing over 5 million geo-located tweets from 39 European countries with the aim of exploring the use of Twitter in transport networks. Two types of transportation systems are considered across the continent: highways and trains. Tweets on the road and on the rail between September 2012 and November 2013 have been identified and the coverage of the total transportation system is analyzed country by country. Differences between countries rise due to the different adoption or penetration rates of geo-located Twitter technology. However, our results show that the penetration rate is not able to explain the full picture regarding differences across counties that may be related to the cultural diversity at play. The paper is structured as follows. In the first section, the datasets are described and the method used to identify tweets on highways and railways is outlined. In the second section, we present the results starting by general features about the Twitter database and then comparing different European countries by their percentage of highway and railway covered by the tweets. Finally, the number of tweets on the road is compared with the Average Annual Daily Traffic (AADT) in France and in the United Kingdom to assess its capacity as a proxy to measure traffic loads.

## Materials and Methods

### 1.1 Datasets

**Figure 1:**
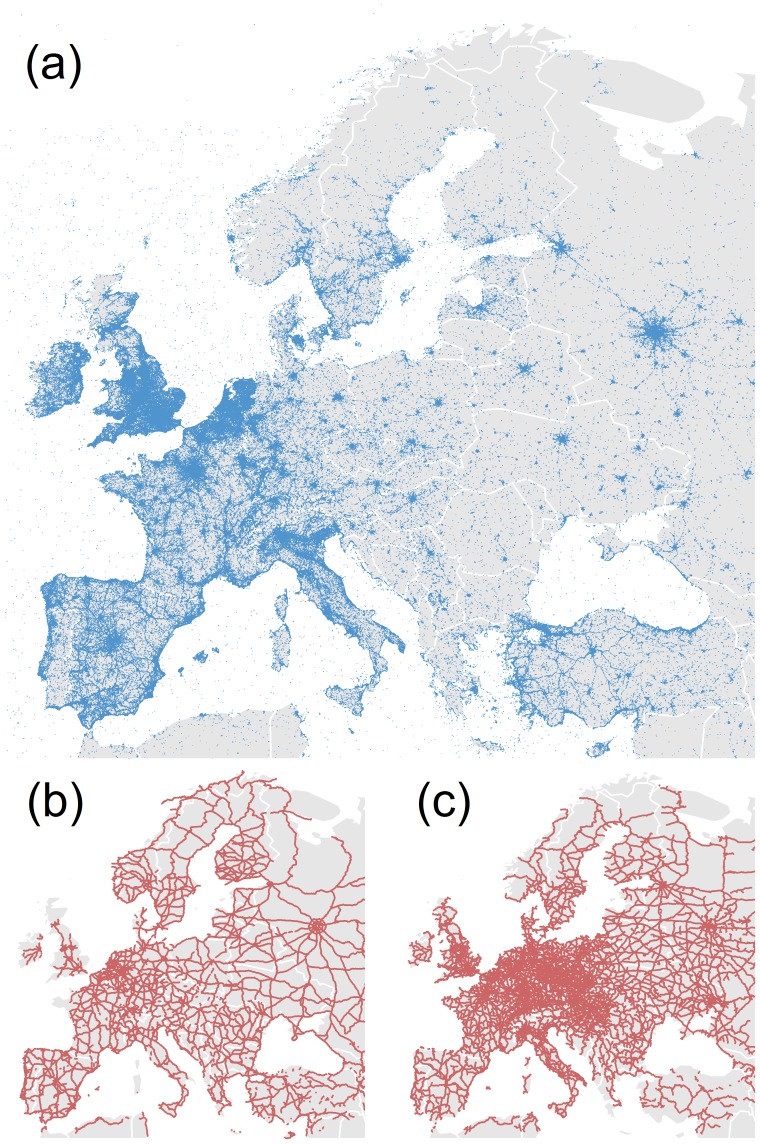
Maps of geo-located tweets, roads and railways. (a) Geo-located tweets on a map. (b) Highway network. (c) Railway network.

The dataset comprehends 5,219,539 geo-located tweets across Europe emitted by 1,477,263 Twitter users in the period going from September 2012 to November 2013. The data was gathered through the general data streaming with the Twitter API [27]. It is worth noting that the tweets are not uniformly distributed, see Figure 1a. Countries of western Europe seem to be well represented, whereas countries of Eastern Europe are clearly under-represented (except for Turkey and Russia).

**Figure 2:**
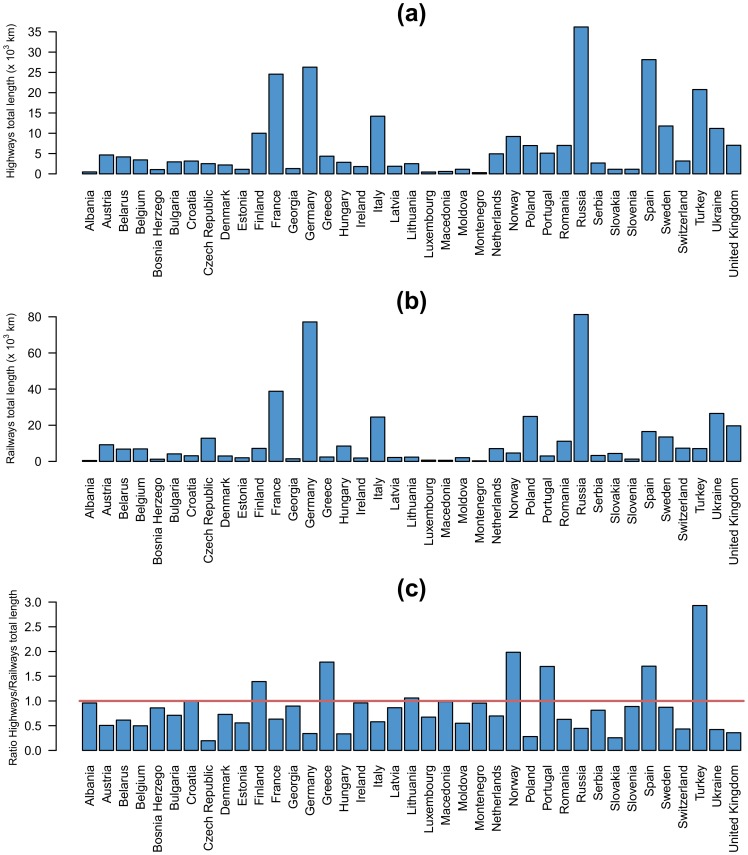
Histograms of total lengths by country of highways and railways. (a) Highways total length by European countries. (b) Railways total length by European countries. (c) Ratio between the total length of highways and that of railways by several European countries. The red line marks the unit ratio.

The highway (both directions) and the railway European networks were extracted from OpenStreetMap [28] (Further details about the OpenStreetMap dataset are given in the Section *OpenStreetMap dataset* and Figure S1 in File S1). Figure 1a, Figure 1b and 1c display maps of geo-located tweets, roads and railways, respectively. A close look at the three maps reveals that while tweets concentrate in cities, there is a number of tweets following the main roads and train lines. In this sense, even roads that go through relatively low population areas can be clearly discerned such as those on Russia connecting the main cities, the area of Monegros in Spain, North of Zaragoza or the main roads in the center of France (see the maps country by country in File S1, Figure S3–S9). Here we analyze in detail the statistics of the tweets posted on the roads and railways and discuss the possibility that they are a proxy for traffic and cultural differences. It is important to stress that we considered only the main highways (motorways and international primary roads), not rural roads, while for railways we considered all the main lines (standard gauge of the considered country). The European highways and railways that we consider have a total length of 274,365 kilometers and 451,475 kilometers, respectively, which have been divided into segments of 10 kilometers each. The histograms of total lengths by country of highways (panel (a)) and railways (panel (b)) are plotted in Figure 2. Russia, Spain, Germany, France and Turkey represent 50% of the highways total length in Europe. While, for the railway, Russia and Germany represent 35% of the total length. Figure 2c shows that most of European countries have a railway network larger than the highway network except for Turkey, Norway, Greece, Spain, Portugal and Finland. In particular, Turkey has a highway system three times larger than the railway network.

### 1.2 Identify the tweets on the road

To identify the tweets on the road/rail, we have considered all the tweets geo-located less than 20 meters away from a highway (both directions) or a railway. Then, each tweet on the road/rail is associated with the closest segment of road/rail. Using this information, we can compute the percentage of road and rail segments covered by the tweets (hereafter called highway coverage and railway coverage). A segment of road or rail is covered by tweets if there is at least one tweet associated with this segment.

### 1.3 Ethics statement

The data analyzed are publicly available as they come from public online sites (Twitter [27] and OpenStreetMap [28]). Furthermore, the Twitter data have been anonymized and aggregated before the analysis that has been performed in accordance with all local data protection laws.

## Results

### 2.4 General features

#### 2.4.1 Penetration rate

**Figure 3:**
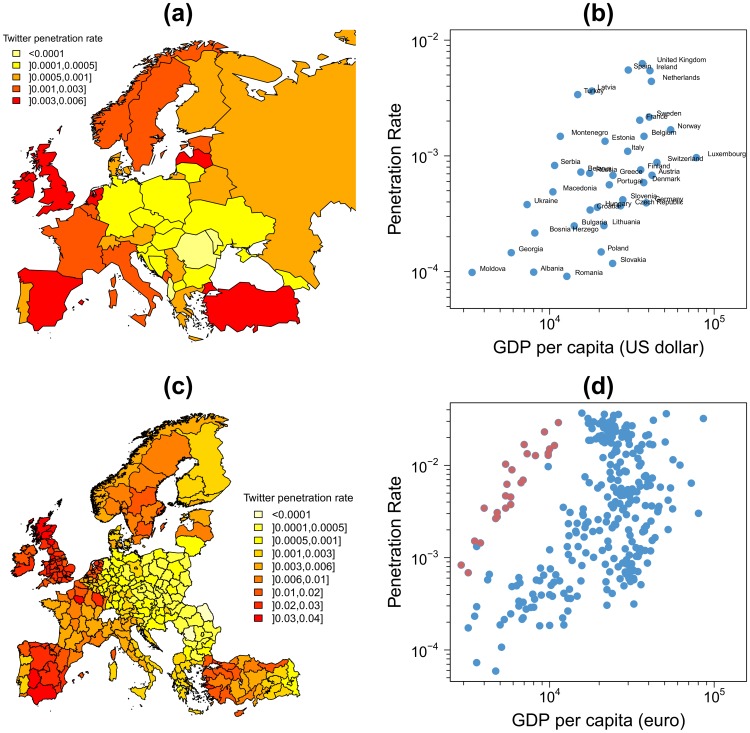
Twitter penetration rate across Europe. (a) and (c) Geolocated Twitter penetration rate across Europe at country level (a) and NUTS 2 level (c). Twitter penetration rate is defined as the ratio between the number of users emitting geo-located tweets in our database and the population in 2012. (b) and (d) Penetration rate as a function of the Gross Domestic Product (GDP) per capita at country level (b) and NUTS 2 level (d). At country level, the figures for the GDP were obtained from the web of the International Monetary Fund [30], correspond to the year 2012 and are expressed in US dollars. At NUTS 2 level, the figures for the GDP were obtained from the web of Eurostat [32], the figures for GDP correspond to the year 2011 and are expressed in euros. Each point represents a country or a NUTS, the red points in (d) represent the NUTS 2 of Turkey.

To evaluate the representativeness of the sample across European countries, the Twitter penetration rate, defined as the ratio between the number of Twitter users and the number of inhabitants of each country, is plotted in Figure 3a. This ratio is not distributed uniformly across European countries. The penetration rate is lower in countries of central Europe. It has been shown in previous studies [20], [24] that the Gross Domestic Product (GDP) per capita (an indicator of the economic performance of a country) is positively correlated with the penetration rate at a world-wide scale. Figure 3b shows the penetration rate as a function of the GDP per capita in European countries. No clear correlation is observed in this case. This fact does not conflict with the previous results since our analysis is restricted to Europe and as shown in [20], in this relationship, countries from different continents cluster together. This means that a global positive correlation appears if countries from all continents are considered but it is not necessarily significant when the focus is set instead on a particular area of the world. To evaluate the robustness of this result we have plotted the penetration rate as a function of the GDP per capita at NUTS 2 level (Figure 3d) and NUTS 3 level (Figure S2 in File S1). The NUTS classification (Nomenclature of territorial units for statistics) is a hierarchical system for dividing up the economic territory of the European Union [29]. It is interesting to note that the correlation between the penetration rate and the GDP per capita remains very low even if we increase the resolution. Actually, geolocated-Twitter penetration rate is evenly distributed internally across most European countries (Figure 3d). One exception is, for instance, Turkey (red dots in Figure 3d), where an important gradient in penetration rate can be observed from Istanbul to the South East. A similar gradient exists for the GDP per capita, and in practice both variables are correlated as also occurs at a world-wide scale (see red points in Figure 3d).

#### 2.4.2 Social network

The penetration rate of geo-located tweets is different across European countries and does not show a clear relation to the GDP per capita of each country. There are several factors that can contribute to this diversity such as the facility of access or prices of the mobile data providers. In addition, generic cultural differences when facing a delicate issue from the privacy perspective such as declaring the precise location in posted messages can be also present. One can then naturally wonder whether these differences extend to other aspects of the use of Twitter or are constraint to geographical issues. One obvious question to explore is the structure of the social network formed by the interactions between users. We extract interaction networks by establishing the users as nodes and connecting a pair of them when they have interchanged a reply. Replies are specific messages in Twitter designed to answer the tweets of a particular user. It can be seen as a direct conversation between two users and as shown in [31] (and references therein) can be related to more intense social relations. A network per country was obtained by assigning to each user the country from which most of his or her geo-located tweets are posted.

**Figure 4:**
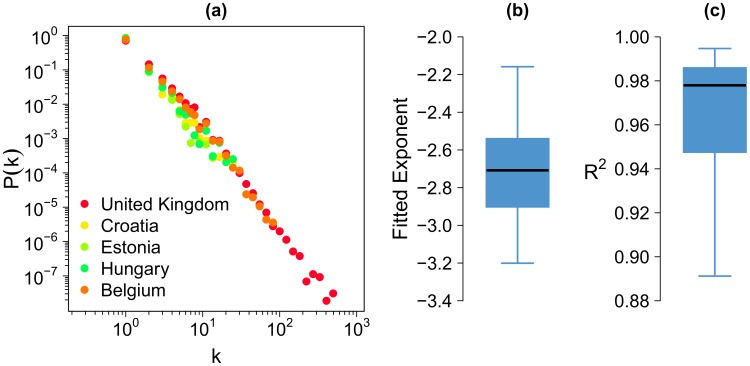
Distribution of the social network's degree. (a) Probability distribution of number of ties of an individual in the social network of 5 countries drawn at random among the 39 case studies. (b) Box plot of the 39 fitted exponent values. (c) Box plot of the *R* ^2^ values. The box plot is composed of the minimum value, the lower hinge, the median, the upper hinge and the maximum value.

Figure 4a shows the distribution of the social network's degree (number of connections per user) of 5 countries (Belgium, Croatia, Estonia, Hungary and the UK) drawn at random among the 39 considered. The slope of these 5 distributions are very similar and can be fitted using a power-law distribution. More systematically, in Figure 4b and Figure 4c we have respectively plotted the box plot of the fitted exponent values obtained for the 39 countries and the box plot of the R^2^ associated with these fits. All the 39 networks have very similar degree distributions, although they show a different maximum degree as a result of the diverse network sizes. These networks are sparse due to the fact that we are keeping only users if they post geo-located tweets and connections only if a reply between two users have taken place. Still and beyond the degree distribution, other topological features such as the average node clustering seems to be quite similar across Europe laying between 0.02 and 0.04 for the most populated countries (where we have more data for the network).

### 2.5 Highway and railway coverage

#### 2.5.1 Dependence between coverage and penetration rate

**Figure 5:**
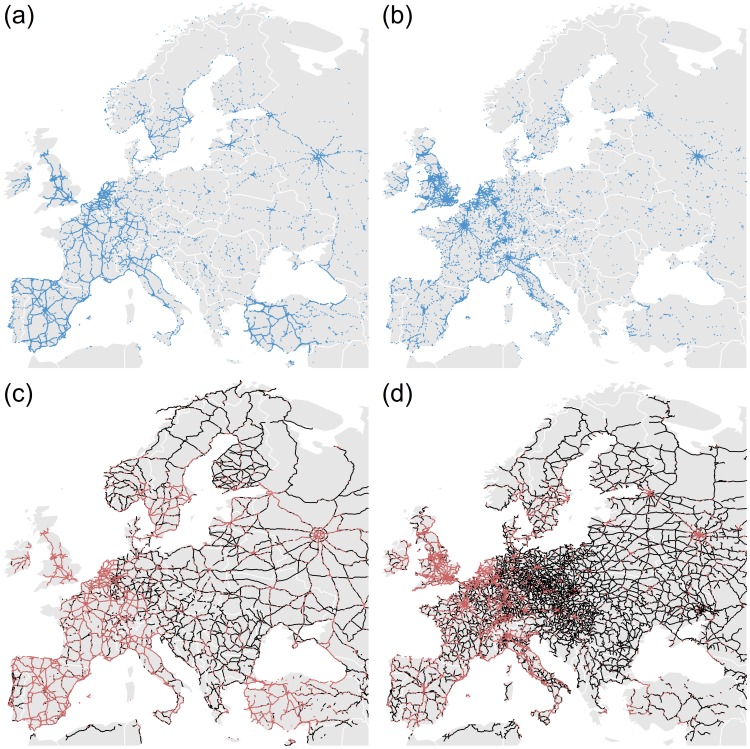
Highway and railway coverages. (a)–(b) Locations of the geo-located tweets on the road (a) and rail (b). (c)–(d) Segments of road (c) and rail (d) covered by the tweets. The red segments represent the segments covered by the tweets.

The percentage of segments (i.e., km) covered by the tweets in Europe is 39% for the highway and 24% for the railway. The highway coverage is better than the railway coverage probably because the number of passenger-kilometers per year, which is the number of passengers transported per year times kilometers traveled, on the rail network is lower. However, the coverage is very different according to the country. Indeed, in Figure 5 we can observe that western European countries have a better coverage than countries of eastern Europe except Turkey and, to a lesser extent, Russia.

**Figure 6:**
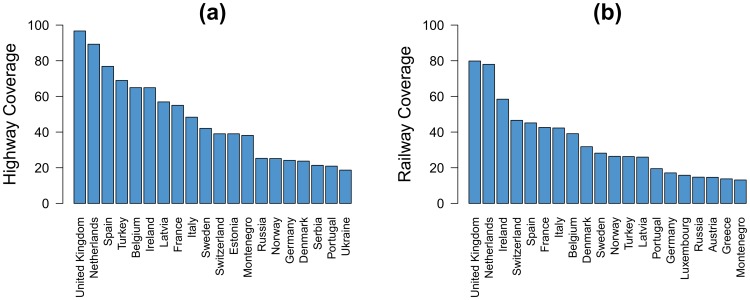
Top 20 of the countries ranked by highway coverage (a) and railway coverage (b).

**Figure 7:**
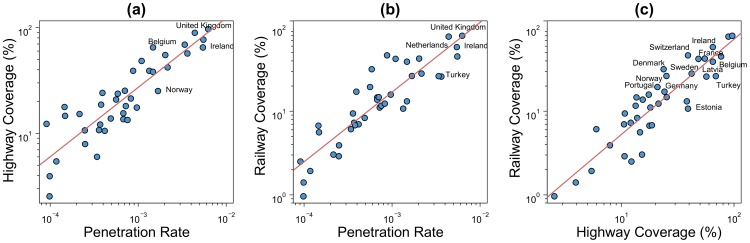
Highway and railway coverages as a function of the penetration rate. (a) Highway coverage as a function of the penetration rate. (b) Railway coverage as a function of the penetration rate. (c) Railway coverage as a function of the highway coverage. Red lines are linear regression curves applied to the log-log plots. This mean that the slope corresponds to the exponents of power-law relationships. The slope in (a) is around 0.6, 0.8 in (b) and 1 (a linear relation) in (c).

Figure 6 shows the top 20 European countries ranked by highway coverage (Figure 6a) and railway coverage (Figure 6b). The two countries with the best highway and railway coverages are the United Kingdom and the Netherlands. The tweets cover 97% of the highway system in UK and 89% in Netherlands. On the other hand, the tweets cover up to 80% of the railway network in the UK and 78% in Netherlands. Inversely, the country with the lowest coverage is Moldavia with a highway coverage of 2.5% and a railway coverage of 1%. The first factor to take into account to understand such differences is the penetration rate. In fact, as it can be observed in Figure 7a and Figure 7b, as a general trend, the coverage of both highway and railway networks is positively correlated with the penetration rate. And, as a consequence, a positive correlation can also be observed between the highway coverage and the railway coverage (Figure 7c). However, these relationships are characterized by a high dispersion around the regression curve. Note that the dispersion is higher than what it can look in a first impression because the scales of the plots of Figure 7 are logarithmic. For the two first relationships the mean absolute error is around 7.5% and for the third one the mean absolute error is around 6.5%. This implies that divergences on the geo-located Twitter penetration does not fully explain the coverage differences between the European countries.

#### 2.5.2 Differences across European countries

**Figure 8:**
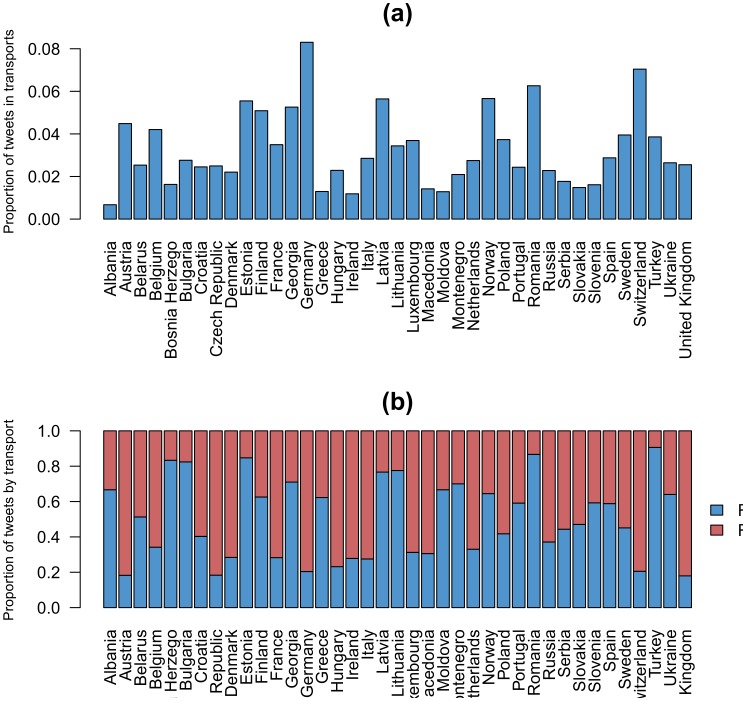
Proportion of tweets in transports. (a) Proportion of tweets on the highway or railway networks by European countries. (b) Proportion of tweets according to the transport network by European countries. The blue color represents the tweets on the road. The red color represents the tweets on the rail. The proportions have been normalized in order to obtain a total proportion of tweets in transport equal to 1.

Disparity in coverage between countries can neither be satisfactorily explained by differences in fares or accessibility to mobile data technology. For example, two countries as France and Spain are similar in terms of highway infrastructure, mobile phone data fares and accessibility, but the geo-located Twitter penetration rates are very different as also are their highway coverage 77% in Spain and 55% in France. Besides penetration rates, divergences in coverage might be the product of cultural differences among European countries when using Twitter in transportation. As it can be observed in Figure 8a, the proportion of tweets geo-located on the highway or railway networks is very different from country to country. In the following, we focus on three examples of countries with similar characteristics in the sense of penetration rates but displaying significant differences in transport network coverage.

#### Ireland and United Kingdom

The most explicit example of the impact of cultural differences on the way people tweet in transports could be given by the Ireland and United Kingdom case studies. Indeed, these two countries have very similar penetration rates but UK has a proportion of tweets in transports more than two times higher than Ireland. Moreover, both highway and railway coverages are one and a half times higher in UK than in Ireland.

#### Turkey and Netherlands

Turkey and Netherlands, which have similar penetration rate, are also an interesting example illustrating how cultural and economical differences may influence coverage. Despite the fact that they both have a high highway coverage, Netherlands has a railway coverage three times higher than Turkey. Turkey is a more extensive and heterogeneous country, including important economic differences across it. Possible economic differences of train and car travelers could be an explanation for the observed disparity in tweets from the roads and trains.

#### Belgium and Norway

For countries having similar penetration rate, the higher the proportion of tweets in transports, the better the coverage. However, some exceptions exist, for example, Norway has a proportion of tweets in transports higher than Belgium but, inversely, Belgium has a highway coverage three times higher than Norway. Given the very extensive highway system of Norway, some of the segments, especially on the North, can have very low traffic, which could be the origin of this difference.

**Figure 9:**
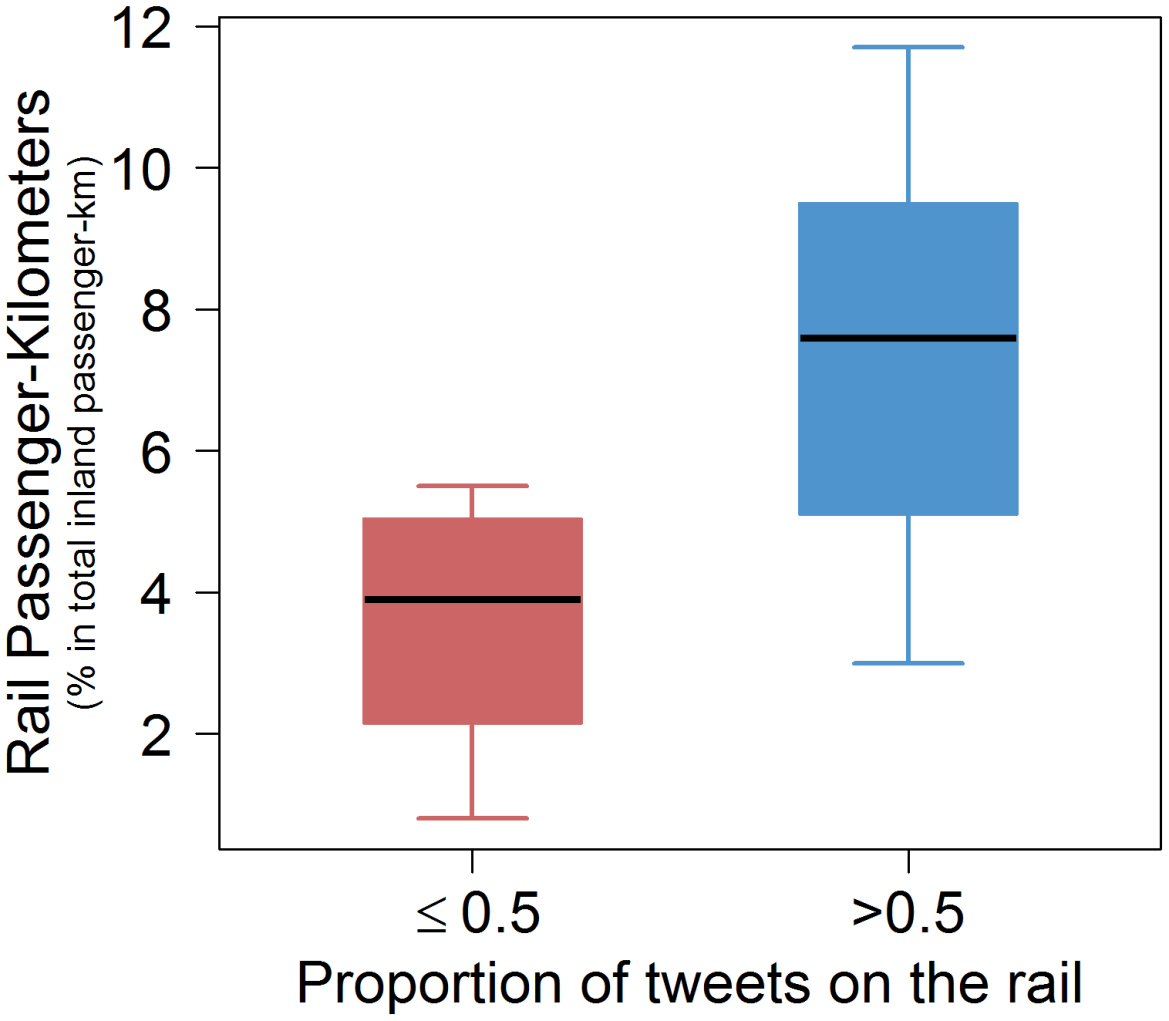
Box plots of the distribution of rail passenger-kilometers expressed in percentage of total inland passenger-kilometers according to the proportion of tweets on the rail among the tweets on the road and rail. The red color for countries having a proportion of tweets on the rail less than 0.5. The blue color for countries having a proportion of tweets on the rail higher than 0.5.

In general, the distribution of tweets according to the transport network is also very different from country to country (Figure 8b) but also region by region. For example, countries from North and Central Europe have a higher proportion of tweets on the road than tweets on the rail than others European countries. This is probably due to difference regarding the transport mode preference among European countries. To check this assumption, we studied the distribution of rail passenger-kilometers in 2011 [32] according to the proportion of tweets on the rail. Figure 9 shows box plots of the distribution of rail passenger-kilometers expressed in percentage of total inland passenger-kilometers according to the proportion of tweets on the rail among the tweets on the road and rail. Globally, the number of rail passenger-kilometers is lower for countries having a low proportion of tweets on the rail, which confirms our assumption.

In the same way, the distribution of rail passenger-kilometers in 2011 can be used to understand why two countries having the same highway coverage might have very different railway coverages. For example, Switzerland and Estonia have the same highway coverage with about 40% of road segments covered by the tweets but the railway coverage is very different, with about 47% of rail segments covered in Switzerland and 11% in Estonia. This can be explained by the fact that in Switzerland trains accounted for 17.6% of all inland passenger-kilometers in 2011 (which was the highest value among European countries in that year) and inversely, in Estonia, trains accounted for 2% of all inland passenger-kilometers (one of the lowest in Europe). More systematically, for each pair of countries having similar highway coverages, we compared the difference between railway coverages and the difference between the percentage of rail passenger-kilometers. First, pair of countries having a highway coverage higher than 20% and an absolute different between their highway coverages lower than 5% are selected. Thus, we have selected 13 pairs of countries with a similar highway coverage. Table 1 displays the difference between the percentage of rail passenger-kilometers and the difference between the railway coverages for these 13 pairs of countries. In 10 out of 13 cases, the differences have the same sign. This fact points towards a possible correlation between traffic levels and tweet coverage.

**Table 1:** Difference between the percentage of rail passenger-kilometers and difference between the railway coverages for each pair of countries having a highway coverage higher than 20% and an absolute different between their highway coverages lower than 5%.

Pair of countries	Difference between the percentage of rail passenger-kilometers	Difference between the railway coverages
Belgium-Turkey	0.13	4.8
Ireland-Belgium	0.19	−4.3
Ireland-Turkey	0.32	0.5
France-Latvia	0.17	5.2
Switzerland-Sweden	0.18	8.1
Sweden-Estonia	0.17	7.5
Switzerland-Estonia	0.36	15.6
Norway-Germany	0.09	−3.6
Danmark-Norway	0.05	4.5
Norway-Portugal	0.07	0.2
Danmark-Germany	0.15	0.9
Portugal-Germany	0.02	−3.8
Danmark-Portugal	0.12	4.7

### 2.6 Average Annual Daily Traffic

To assess more quantitatively this hypothetical relation between the number of vehicles and the number of tweets on the road, we compared the number of tweets and the Average Annual Daily Traffic (AADT) on the highways in United Kingdom in 2012 [33] and in France in 2011 [34]. The AADT is the total number of vehicle traffic of a highway divided by 365 days. The number of highway segments for which the AADT was gathered is 877 in UK and 1974 in France. The average length of these segments is 3.6 kilometers in UK and 5.8 in France. As in the previous analysis, the number of tweets associated with a segment was computed by identifying all the tweets geo-located at less than 20 meters away from the segment.

**Figure 10:**
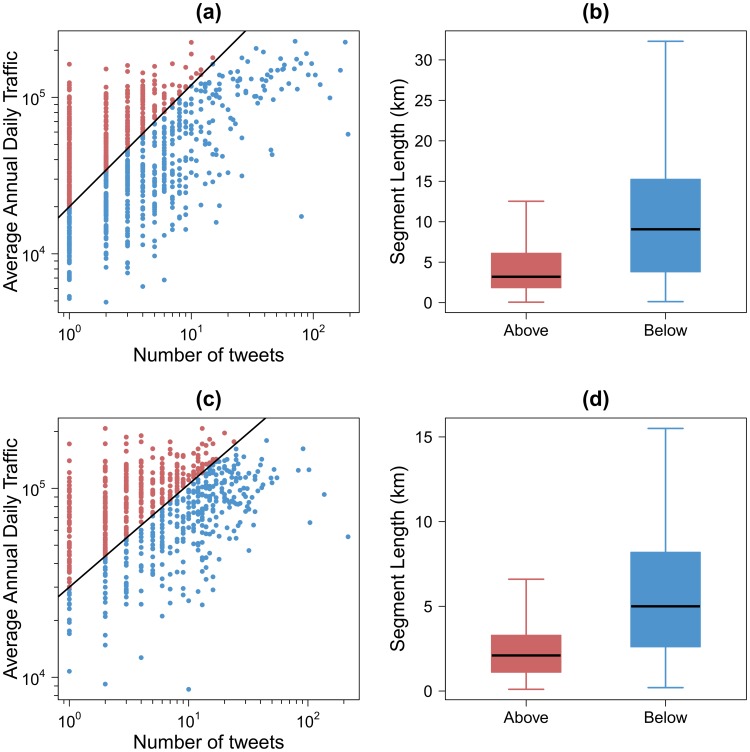
Comparison between the Average Annual Daily Traffic and the number of tweets on the road. (a)–(c) Comparison between the Average Annual Daily Traffic and the number of tweets on the highways in France (a) and United Kingdom (b). Points are scatter plots for each highway segments. (b)–(d) Box plots of the highway segment length according to the type of segment in France (b) and United Kingdom (d). The red color for the points above the black line and the blue color for the points below the black line.

Figure 10a and 10c shows a comparison between the AADT and the number of tweets on the road for both case studies. There is a positive correlation between the AADT and the number of tweets on the road but the Pearson correlation coefficient values are low, around 0.5 for the France case study and around 0.3 for the UK case study. This can be explained by the large number of highway segments having a high AADT but a very low number of tweets. To understand the origin of such disagreement between tweets and traffic, we have divided the segments into two groups: those having a high AADT and a very low number of tweets (red points) and the rest (blue points). These two types of segments have been separated using the black lines in Figure 10a and 10c. Figure 10b and 10d show the box plots of the highway segment length in kilometer according to the segment type for both case studies. It is interesting to note that the segments having a high AADT and a low number of tweets are globally shorter than the ones of the other group. Indeed, according to the Welch two sample t-test [35] the average segment length of the first group (5.2 km in France and 2.5 in UK) is significantly lower than the one of the second group (11.3 km in France and 5.9 in UK). In both cases the *p-value* is less than 10^−5^. Given a similar speed one can assume that the shorter the road segment is, the lower time people have to post a tweet. Other factors that may influence this result is the nature of the segments, rural vs urban, and the congestion levels that can significantly alter the time spent by travelers in the different segments.

## Discussion

In this work, we have investigated the use of Twitter in transport networks in Europe. To do so, we have extracted from a Twitter database containing more than 5 million geo-located tweets posted from the highway and the railway networks of 39 European countries. First, we show that the countries have different penetration rates for geo-located tweets with no clear dependence on the economic performance of the country. Our results show, as well, no clear difference between countries in terms of the topological features of the Twitter social network. Dividing the highway and railway systems in segments, we have also studied the coverage of the territory with geo-located tweets. European countries can be ranked according to the highway and railway coverage. The coverages are very different from country to country. Although some of this disparity can be explained by differences in penetration rate or by the use of different transport modalities, a large dispersion in the data still persist. Part of it could be due to cultural differences among European countries regarding the use of geo-located tools. Finally, we explore whether Twitter can be used as a proxy to measure of traffic on highways by comparing the number of tweets and the Average Annual Daily Traffic (AADT) on the highways in United Kingdom and France. We observe a positive correlation between the number of tweets and the AADT. However, the quality of this relationship is reduced due to the short character of some AADT highway segments. We conclude that the number of tweets on the road (train) can be used as a valuable proxy to analyze the preferred transport modality as well as to study traffic congestion provided that the segment length is enough to obtain significant statistics.

## Supporting Information

File S1 **Contains Figures S1–S9.**
(PDF)
