# Supplementary material for: Tweets on the Road
Source: PLoS One. 2014 Aug 20;9(8):e105407. doi: 10.1371/journal.pone.0105407 (PMC4139377; doi:10.1371/journal.pone.0105407)
Supplement: File S1 — Contains Figures S1–S9. (PDF) [file pone.0105407.s001.pdf]

Supporting Information: Tweets on the road

Maxime Lenormand[1], Antònia Tugores[1], Pere Colet[1] José J. Ramasco[1],
1 Instituto de Física Interdisciplinar y Sistemas Complejos IFISC (CSIC-UIB), Palma de Mallorca, Spain

Email address of corresponding author: maxime@ifisc.uib-csic.es

OpenStreetMap dataset

Both railway and highway networks were extracted from OpenStreetMap using *osmfilter*[1] which is a software used to filter OpenStreetMap data files for specific tags. To extract the railway network from OpenStreetMap the tag *"railway=rail"* has been used. This tag corresponds to the largest railway classification (in the standard gauge for the country or state). For the highway network, we used three different tags because road classifications are very different from country to country. In order to extract a homogeneous European highway network we used the tag "highway=motorway" for Western Europe and the tags "highway=trunk" and "highway=primary and int_ref=E*" for Eastern Europe. As it can be observed in Figure S1 the resulting network is quite homogeneous across European countries.

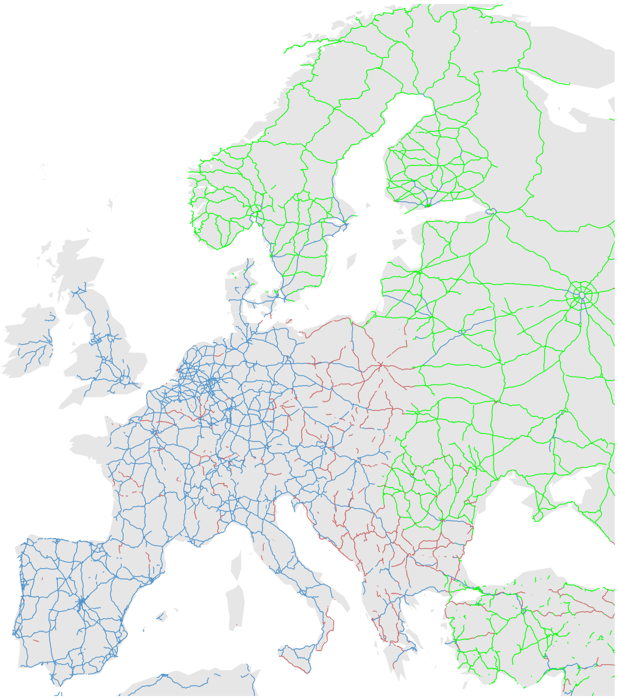

Figure S1. Highway network. Blue segments represent the road segments extracted using the tag "highway=motorway". Green segments represent the road segments extracted using the tag "highway=trunk". Red segments represent the road segments extracted using the tag "highway=primary and int_ref=E*".

[1] http://wiki.openstreetmap.org/wiki/Osmfilter

Supplementary Figures

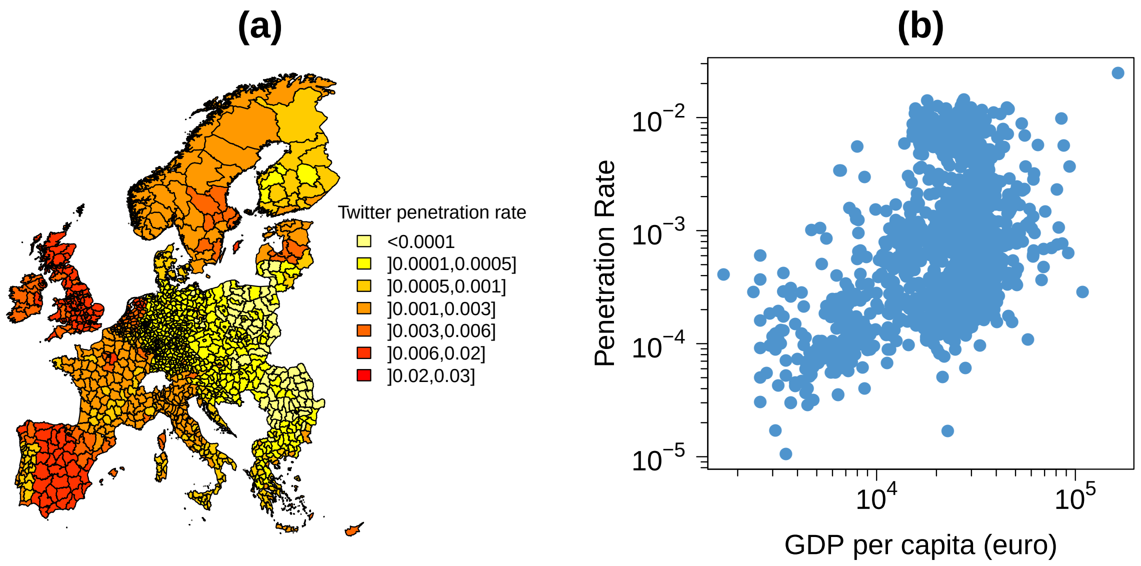

Figure S2. (a) Geolocated Twitter penetration rate across Europe at NUTS 3 level. Twitter penetration rate is defined as the ratio between the number of users emitting geo-located tweets in our database and the population in 2012. (b) Penetration rate as a function of the Gross Domestic Product (GDP) per capita at NUTS 3 level. Data were obtained from the web of Eurostat, the figures for GDP correspond to the year 2011 and are expressed in euros.

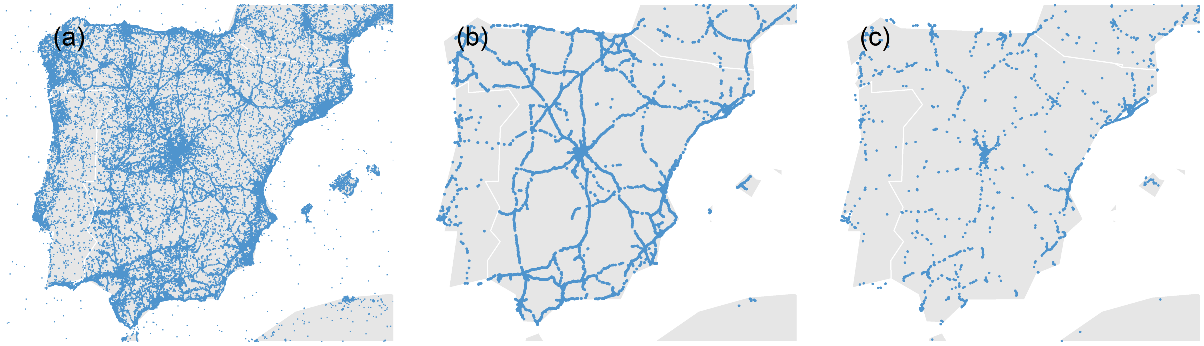

Figure S3. Locations of the geo-located Tweets (a) on the road (b) and on the rail (c) in Spain.

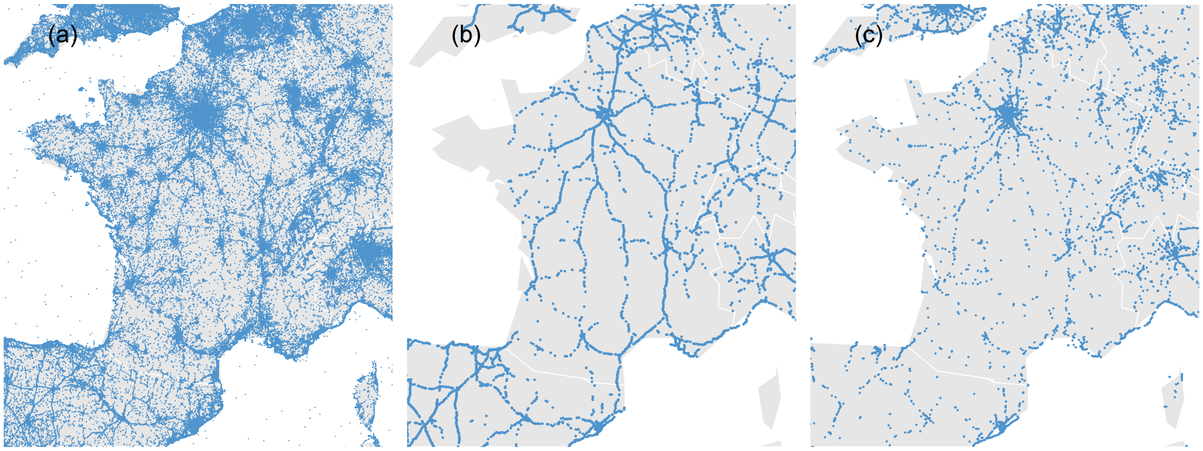

Figure S4. Locations of the geo-located Tweets (a) on the road (b) and on the rail (c) in France.

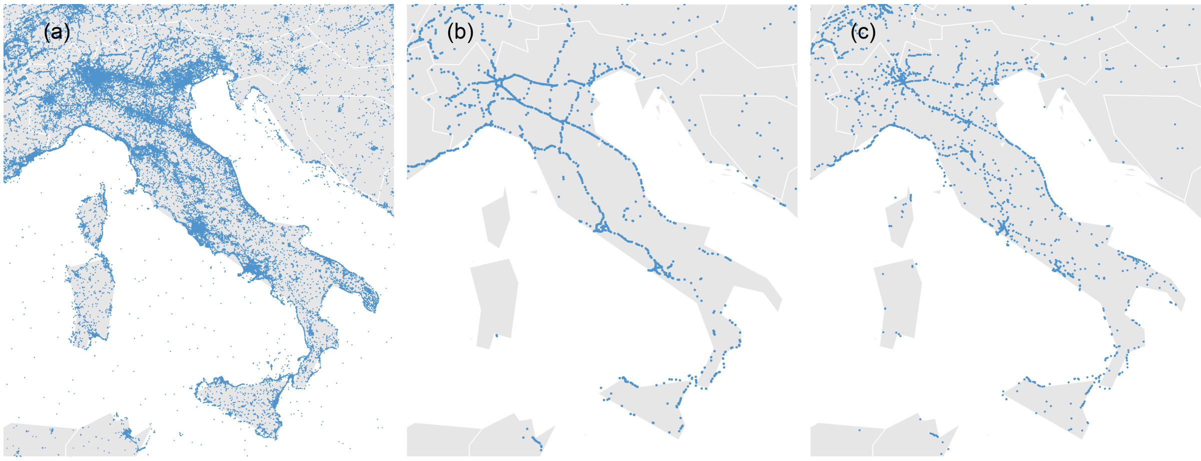

Figure S5. Locations of the geo-located Tweets (a) on the road (b) and on the rail (c) in Italy.

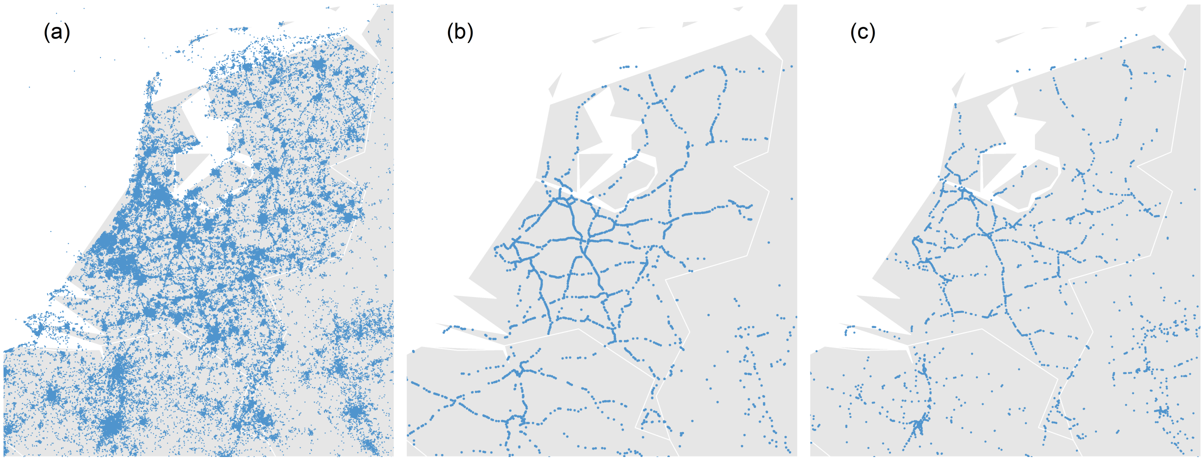

Figure S6. Locations of the geo-located Tweets (a) on the road (b) and on the rail (c) in Netherlands.

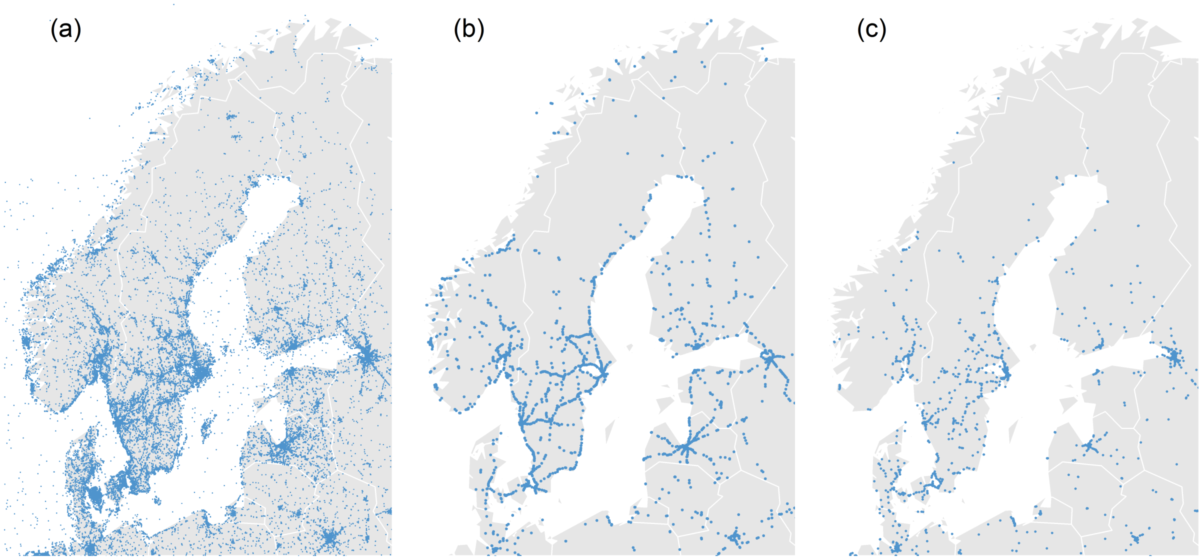

Figure S7. Locations of the geo-located Tweets (a) on the road (b) and on the rail (c) in Norway.

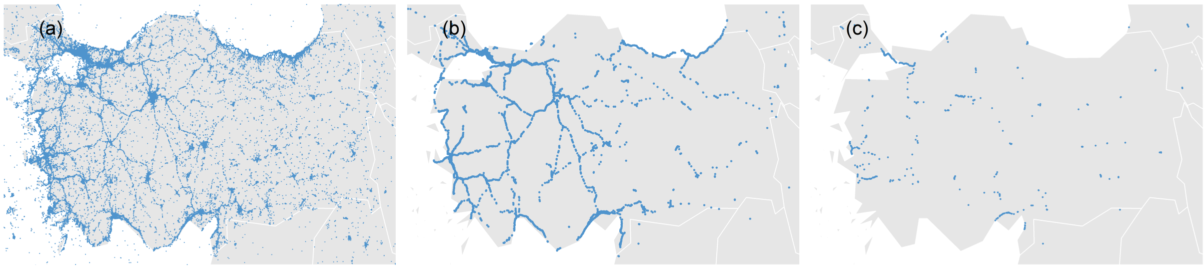

Figure S8. Locations of the geo-located Tweets (a) on the road (b) and on the rail (c) in Turkey.

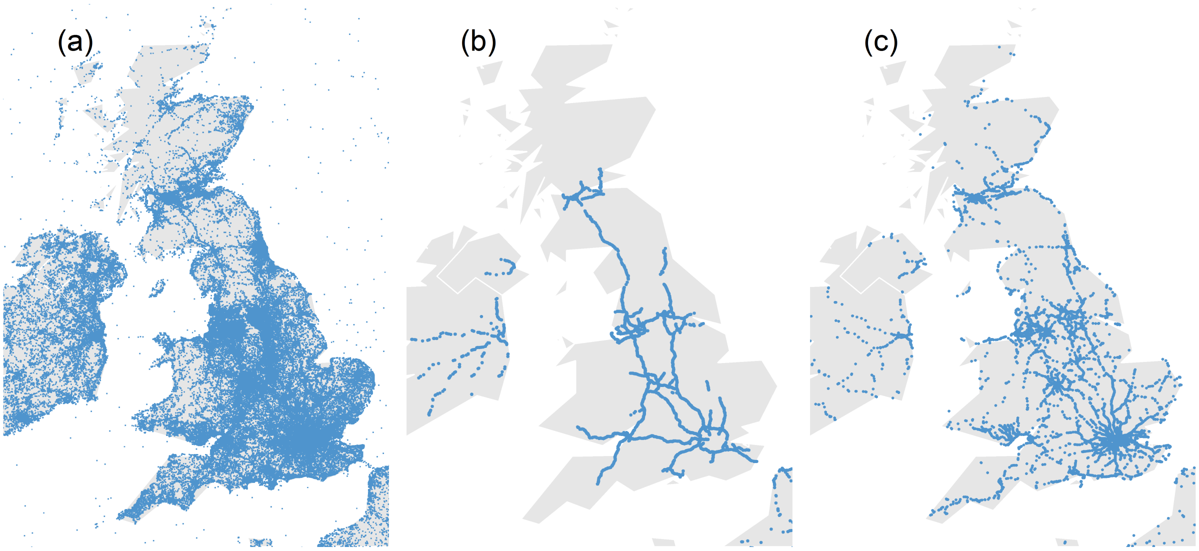

Figure S9. Locations of the geo-located Tweets (a) on the road (b) and on the rail (c) in United Kingdom.
